# Phenotypic and functional characterization of the major lymphocyte populations in the fruit-eating bat *Pteropus alecto*

**DOI:** 10.1038/srep37796

**Published:** 2016-11-24

**Authors:** Julia María Martínez Gómez, Pravin Periasamy, Charles-Antoine Dutertre, Aaron Trent Irving, Justin Han Jia Ng, Gary Crameri, Michelle L. Baker, Florent Ginhoux, Lin-Fa Wang, Sylvie Alonso

**Affiliations:** 1 Department of Microbiology and Immunology, Yong Loo Lin School of Medicine, National University of Singapore, Singapore; 2 Immunology programme, Life Sciences Institute, Yong Loo Lin School of Medicine, National University of Singapore, Singapore; 3 Programme in Emerging Infectious Disease, Duke-NUS Medical School, Singapore; 4 Singapore Immunology Network (SIgN), Agency for Science, Technology and Research (ASTAR), Singapore; 5 CSIRO, Health and Biosecurity Business Unit, Australian Animal Health Laboratory, Geelong, Australia

## Abstract

The unique ability of bats to act as reservoir for viruses that are highly pathogenic to humans suggests unique properties and functional characteristics of their immune system. However, the lack of bat specific reagents, in particular antibodies, has limited our knowledge of bat’s immunity. Using cross-reactive antibodies, we report the phenotypic and functional characterization of T cell subsets, B and NK cells in the fruit-eating bat *Pteropus alecto*. Our findings indicate the predominance of CD8^+^ T cells in the spleen from wild-caught bats that may reflect either the presence of viruses in this organ or predominance of this cell subset at steady state. Instead majority of T cells in circulation, lymph nodes and bone marrow (BM) were CD4^+^ subsets. Interestingly, 40% of spleen T cells expressed constitutively IL-17, IL-22 and TGF-β mRNA, which may indicate a strong bias towards the Th17 and regulatory T cell subsets. Furthermore, the unexpected high number of T cells in bats BM could suggest an important role in T cell development. Finally, mitogenic stimulation induced proliferation and production of effector molecules by bats immune cells. This work contributes to a better understanding of bat’s immunity, opening up new perspectives of therapeutic interventions for humans.

In recent years bats have been recognized as important reservoirs of highly pathogenic viruses for human and other animals such as SARS coronavirus, Hendra, and Nipah viruses^1^,^2^. Bats have also been proposed as potential reservoir for Ebola^3^ and MERS^4^, although direct evidence is still lacking. How do bats cope with viruses that have a high mortality rate in humans without getting sick? How have they evolved to co-exist with such viruses? Understanding the bats immune system should help address this question and might open a door to new therapeutic interventions for humans.

Immunity in mammals comprises two main arms, namely the innate and the adaptive immunity, that interact with each other to generate optimal responses against pathogens. The innate immunity provides immediate nonspecific host defenses and is highly conserved among species; whereas the adaptive immunity is restricted to higher organisms and involves antigen-specific T and B cells^5^. Pathogen-derived antigens are presented by antigen presenting cells (APC) such as macrophages and dendritic cells (DC) to antigen-specific T and B cells in secondary lymphoid organs such as lymph nodes, spleen and various mucosa-associated lymphoid tissues (MALT)^6^. The antigen-specific T or B cells get then activated, proliferate and migrate to the site of infection where they fight the invading pathogen through antibody production and/or direct killing of the infected cells.

The effector T lymphocyte population can be broadly divided into two subsets, namely T helper cells (Th) and cytotoxic T cells (CTL), which are identified by the expression of CD4 and CD8 molecules on their surface, respectively^7^. Upon activation CD4^+^ Th cells produce cytokines, which are essential to help recruit and further activate/differentiate other immune cell types^8^. For example, CD4^+^ Th are critical for B cell differentiation into antibody producing cells (plasma cells) and antibody isotype switching^9^. CD8^+^ T cells also produce cytokines upon activation but in addition release cytolytic molecules such as granzymes and perforin that can directly lyse infected cells, making this T cell subset absolutely critical to fight intracellular infections, in particular viral infections. CD8^+^ T cells also display antitumor activity^10^,^11^.

The black flying fox *Pteropus alecto* belongs to the genus *Pteropus* from the megabat suborder, Megachiroptera, and is among the largest bats in the world. Native from Australia, Papua New Guinea, and Indonesia, this fruit-eating bat is not a threatened species, unlike other bats species. In 1994, *P. alecto* was found to be the natural reservoir for Hendra Virus (HeV), and responsible for spillover into horses in Australia, causing a severe respiratory disease in these animals^12^. Due to the importance of horse races in Australia, research on *P. alecto* has received a lot of attention and strong government (Australia) support. In addition, *P. alecto* bats have been found to harbor other virus species potentially pathogenic to humans, including Lyssavirus, closely related to rabies virus^13^, previously unknown paramyxoviruses^14^ as well as a novel betacoronavirus^15^. Serological evidence of infection with Menangle virus (MenV) in Pteropus spp. in Australia was also reported in 2008^16^.

Building on the substantial knowledge (mostly derived from genome sequence analysis) and tools (handful of cell lines and specific antibodies) available on *P. alecto*, we focused on this bat species to study its immune system. Several studies have suggested the importance of innate immunity in bats in their ability to control virus infection^17^,^18^,^19^. This was supported by the broad expression of IRF7 mRNA transcripts involved in the regulation of the interferon (IFN) responses, in various tissues from *P. alecto*. In addition, IRF7 siRNA knockdown resulted in enhanced viral replication in a bat cell line^17^. Furthermore, the constitutive expression of three *IFN-α* genes in *P. alecto* tissues, which is expected to turn on the cell antiviral state, has also been linked to the ability of bats to coexist with pathogenic viruses^19^.

In contrast, the bat adaptive immunity and its importance in controlling viral infections have been less studied. Recent transcriptome studies from three different bat species have provided evidence that genes involved in adaptive immunity in other species are conserved in bats^20^,^21^,^22^,^23^. These genes include MHC class I and II molecules, T cell receptors and co-receptors such as CD3, CD4, CD8 and CD28, as well as B cell specific markers such as CD19, CD22, CD72 and immunoglobulins. However, the characterization of bat immune cells has not been reported and this is likely due to the lack of specific reagents, in particular, antibodies. While raising monoclonal antibodies specific to bat protein markers represents the best approach, it is nevertheless time consuming and costly. In contrast, cross-reactive antibodies raised against the same targets in other mammals (in particular mouse and human) may offer a cheaper and faster alternative. Using cross-reactive antibodies, flow cytometry and fluorescence *in situ* hybridization (Flow-FISH) technologies we provide here the first phenotypic and functional characterization of the main adaptive immune cell populations in the black flying fox *P. alecto*.

## Results

### Protein sequence alignment

Using the *P. alecto* genome Ensembl database, the amino acid sequence of major lymphocyte surface markers, cytokines and transcriptional factors was aligned with that of their human and mouse counterparts (Table 1). Overall, the identity ranged from 44–95% with higher percentages systematically found between *P. alecto* and human compared to *P. alecto* and mouse (Table 1). Furthermore, the amino acid sequence of intracellular molecules such as transcription factors Gata3, T-bet and Eomes was highly conserved between bats and human/mouse with sequence identity ranging from 88–95%, whereas it was lower for the surface markers (44–78%). High sequence identity was also found between bat TNF and IL-10, and their human counterparts (88 and 83%, respectively).

### Identification of the major lymphocyte cell populations using cross-reactive antibodies

To assess the cross reactivity of anti-human/mouse antibodies with bat ortholog proteins, we tested 47 commercially available antibodies (Table S1). Among which only 9 displayed cross-reactivity by flow cytometry with *P. alecto* lymphocytes. Interestingly, among these 9 cross-reactive antibodies, only 3 target surface molecules (MHCII, CD44 and CD11b), whereas the remaining 6 target intracellular molecules including the intracellular domain of CD3, transcription factors (T-bet, Gata-3 and Eomes), IL-10 and TNF cytokines (Table S1). This observation correlates well with the higher degree of sequence conservation between bats and human/mouse for intracellular molecules (Table 1). It is worth to note that although the transcription factors Foxp3 and RORγt, expressed by CD4^+^ T regulatory cells (Treg) and CD4^+^ Th17 cells respectively in human and mice, were also highly conserved in *P. alecto*, identification of cross-reactive antibodies against these molecules was unsuccessful.

**Figure 1:**
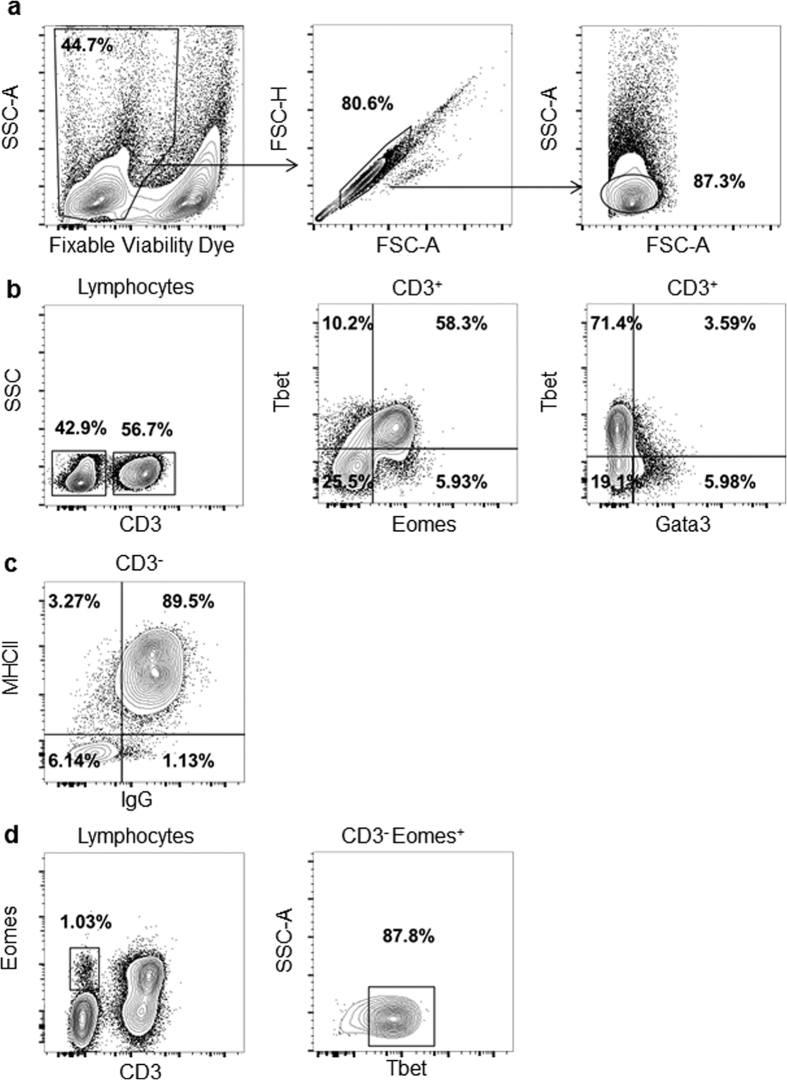
Strategy for immunophenotyping of lymphocytes in *P. alecto.* Bat splenocytes were stained with cross-reactive antibodies and analysed by FACS. (**a**) Gating strategy based on live cells, singlets and lymphocyte region. (**b**) CD3^+^ cell population analysed for the expression of transcription factors Gata3, Tbet and Eomes. (**c**) Strategy to identify B cells based on CD3^−^ MHCII^+^ and IgG^+^ staining. (**d**) Strategy to identify NK cells based on CD3^−^, Eomes^+^ and Tbet^+^ staining. One set of data obtained from one bat spleen is shown, and is representative of 4 different bat spleens analyzed.

Importantly, an antibody targeting the cytoplasmic domain of human CD3 molecule, which has also been reported to be highly cross-reactive with CD3 from other mammals (Table S1), cross-reacted with its bat counterpart, thus allowing identification of the T cell population in *P. alecto*. However, since human/mouse antibodies failed to detect the major lymphocyte surface markers CD4 and CD8 from bat lymphocytes, a strategy based on the expression of transcription factors was adopted to further characterize the T cell subsets. Human and mouse T cell subsets express different transcription factors upon differentiation into effector cells^24^,^25^,^26^. As such, Gata3 has been reported to be the master regulator driving the development of the CD4^+^ Th2 subset, whereas the CD4^+^ Th1 subset expresses predominantly Tbet. As for the CD8^+^ T cell population, co-expression of Tbet and Eomes was described, although Tbet is expressed at an earlier stage than Eomes during the development into effector cells and gets down-regulated later during memory formation^27^,^28^. Based on the assumption that similar T cell subsets with similar expression pattern of transcription factors exist in bats, we devised the following flow cytometry strategy. Total bat splenocytes were first gated on live single cells, excluding doublets (Fig. 1a). The total lymphocyte population was further defined by its forward and side laser light scatter properties (Fig. 1a). Using cross-reactive anti-CD3 antibody, the CD3^+^ lymphocyte population was then identified and selected (Fig. 1b). This cell population stained negative for IgG and CD11b, specific markers for B cells and myeloid cells, respectively, further supporting that these CD3^+^ cells are very likely T lymphocytes (Fig. S1a–c). Tbet, Eomes and Gata3-specific staining of the CD3^+^ population indicated a prominent (58%) Tbet^+^ Eomes^+^ double positive population in this particular bat spleen, which may correspond to CD8^+^ effector T cells (Fig. 1b). Tbet^+^ Eomes^−^ (that could represent the CD4^+^ Th1 cell population or early activated CD8^+^ T cells) and Tbet^−^ Gata3^+^ population (CD4^+^ Th2 cells) were also readily detected although the percentages of both cell populations were relatively low compared to the Tbet^+^ Eomes^+^ CD3^+^ lymphocytes (Fig. 1b).

In addition to T cell subsets, B lymphocytes could be identified by gating on CD3^−^ cells and using a commercial anti-bat IgG antibody combined with cross-reactive anti-MHCII staining. Around 90% of the CD3^−^ lymphocytes stained positive for both IgG and MHCII (Fig. 1c).

Finally, it has been well established that Tbet and Eomes are also essential transcription factors for terminal maturation and homeostasis of human and mouse natural killer (NK) cells^29^,^30^. Consistently, a CD3^−^ Tbet^+^ Eomes^+^ cell population was detected in bat spleen that could be regarded as NK cells (Fig. 1d). The percentage of this cell population among the lymphocyte region (~1–2%) was comparable to that reported for mouse spleen^31^,^32^. Since none of the human/mouse antibodies tested cross-reacted with bat surface markers of NK cells, the differential expression of these transcription factors is for now the only means to identify this immune cell population.

### Identification of the CD4^+^ and CD8^+^ lymphocyte populations by prime flow technology

**Figure 2:**
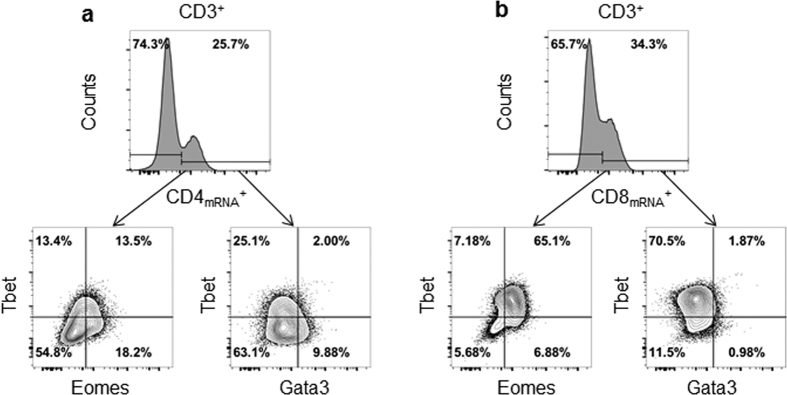
Detection of T cell subsets by combined Flow-FISH and antibody staining. Bat splenocytes were stained with CD3, Tbet, Eomes and Gata3 antibodies followed by *in-situ* hybridization with probes specific for CD4_mRNA_ (**a**) and CD8_mRNA_ (**b**). One data set is shown and is representative of 2 independent experiments performed with 2 different bat spleens.

Since CD4 and CD8 antibody staining failed, PrimeFlow technology was employed, which uses Flow-FISH that allows detection by flow cytometry of specific genes expression at the mRNA level. Thus, simultaneous and combined detection by flow cytometry of protein (using antibodies) and mRNA (using Flow-FISH) at a single cell level is possible^33^. Here, bat splenocytes were stained with anti-CD3, anti-Eomes, anti-Tbet and anti-Gata3 antibodies followed by *in situ* hybridization specific to CD4 and CD8 mRNA. Results indicated that 34% and 25% of the CD3^+^ cells were CD8_mRNA_^+^ and CD4_mRNA_^+^ respectively (Fig. 2). The fact that these percentages added up to 60% only of the total CD3^+^ population suggests lower sensitivity of flow-FISH assay compared to antibody staining. In addition, since the specificity and sensitivity for each target mRNA vary from one probe to another; direct quantitative comparison of different cell populations identified by Flow-FISH may be biased. However, it is possible to analyze sub-populations within a specific cell subset identified by Flow-FISH. Here, the combined Flow-FISH and antibody detection approach reveals that majority (75%) of the CD3^+^ CD8_mRNA_^+^ cells co-expressed Tbet and Eomes, and only about 1% expressed Gata3 (Fig. 2b). This result thus validates our FACS strategy based on the differential expression of Eomes, Tbet and Gata3 to identify the CD8^+^ T cell population. In addition, comparable percentages of CD4_mRNA_^+^ and CD8_mRNA_^+^ cells that were Tbet^+^ Eomes^−^ were observed (13.4 and 10.6%, respectively). This observation thus supports that bat CD3^+^ Tbet^+^ Eomes^−^ cells are likely to represent the CD4^+^ Th1 subset as well as early activated CD8^+^ T cells. Among the CD4_mRNA_^+^ cells, another 12% expressed Gata3 (which could be CD4^+^ Th2 cells), and 13% co-expressed Tbet and Eomes. However, the greatest majority of CD4_mRNA_^+^ cells were Tbet^−^ Gata3^−^ Eomes^−^ triple negative, which could represent naïve CD4^+^ T cells or other subsets that could not be identified due to the lack of cross-reactive antibodies.

Together, these results indicated that majority of CD8_mRNA_^+^ T cells are Tbet^+^ Eomes^+^ double positive and do not express Gata3. Instead, majority of CD4_mRNA_^+^ T cells are Tbet ^−^ Gata3^−^ Eomes^−^ triple negative. However, a substantial proportion of CD4_mRNA_^+^ T cells express Gata3, or Tbet and Eomes.

### Differential expression of transcription factors in CD3^+^ T cells in various organs of *P. alecto*

**Figure 3:**
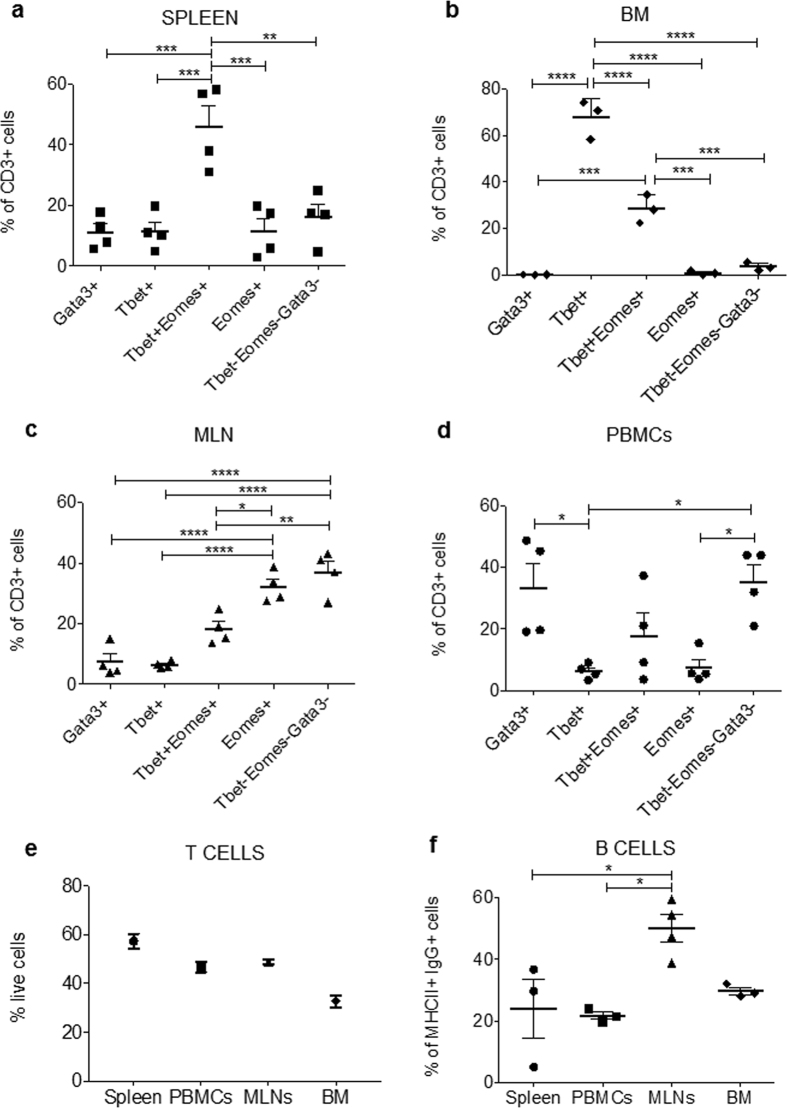
Immune cell populations in *P. alecto* spleen, lymph nodes, blood and bone marrow. Percentage of different CD3^+^ cell subsets based on expression of transcription factors T-bet, Eomes and Gata3 in bat spleen (**a**), mediastinal lymph nodes (MLNs) (**b**), peripheral blood mononuclear cells (PBMCs) (**c**) and bone marrow (BM) (**d**). Percentage of T cells (based on CD3^+^ cells) in spleen, MLN, PBMCs and BM (**e**). Percentage of B cells (based on MHCII^+^ and IgG^+^ double positive cells) in spleen, MLN, PBMCs and BM (**f**). Spleen, MLN and PBMCs from 3–5 bats were analysed. Individual data are represented together with the mean (horizontal bar) and SEM. *p ≤ 0.05, **p ≤ 0.01, ***p ≤ 0.001, ****p ≤ 0.0001 based on 1-way ANOVA with Bonferroni’s post-test.

Guided by the FACS and Flow-FISH data described above, we further investigated the various CD3^+^ T cell subsets in spleen, bone marrow, mediastinal lymph nodes (MLN) and peripheral blood mononuclear cells (PBMCs) from 3–4 wild-caught *P. alecto* bats. Together, the data revealed differential expression patterns of the transcription factors Tbet, Eomes and Gata3 in various organs, suggesting a differential representation and/or activation status of T cell subsets in these organs (Fig. 3). Predominance of Tbet^+^ Eomes^+^ double positive cells (largely representing the CD8+ T cell population) was confirmed in the spleen from the 4 bats examined (Fig. 3a). In sharp contrast, the T cell subsets from the bone marrow comprises of ~70% Tbet^+^ single positive T cells (assumed to be CD4^+^ Th1 cells) and ~30% Tbet^+^ Eomes^+^ double positive T cells (CD8^+^ T cells) (Fig. 3b). The predominant populations in the MLNs were Tbet^−^ Eomes^−^ Gata3^−^ cells (assumed to be CD4^+^ T cells) and Eomes^+^ single positive T cells, which have been described as memory CD8^+^ T cells in other mammals^34^ (Fig. 3c). In PBMCs, Tbet^−^ Eomes^−^ Gata3^−^ triple negative as well as Gata3^+^ single positive T cells (both subsets assumed to be CD4^+^ T cells) dominated (Fig. 3d). Together, the data suggest a predominance of CD8^+^ T cells in the bat spleen, whereas bone marrow and PBMCs display majority of CD4^+^ T cells and MLNs harbor similar proportions of CD4^+^ and CD8^+^ T cells.

Furthermore, interestingly, whereas the percentage (50%) of CD3^+^ T cells among the total leukocytes in bats PBMCs and MLNs is similar to that found in mice at steady state, it reaches 60% in the spleen, which is twice the percentage found in mice (Fig. 3e). Even more strikingly, we found that over 30% of the total leukocytes present in bats bone marrow are CD3^+^ T cells, which is far greater than the percentage described in human and mouse bone marrow which typically ranges between 4% and 8%^35^. This finding suggests that in bats the bone marrow may play a major role in T cell hematopoiesis in contrast to other mammals where the thymus usually fulfills this function; this hypothesis is consistent with the fact that we were unable to find the thymus in these wild caught bats.

In addition, the percentage of B cells based on detection of MHCII and IgG at the cell surface, appeared significantly higher in the MLNs compared to PBMCs and spleen (Fig. 3f).

### Expression of MHCII on T cells

**Figure 4:**
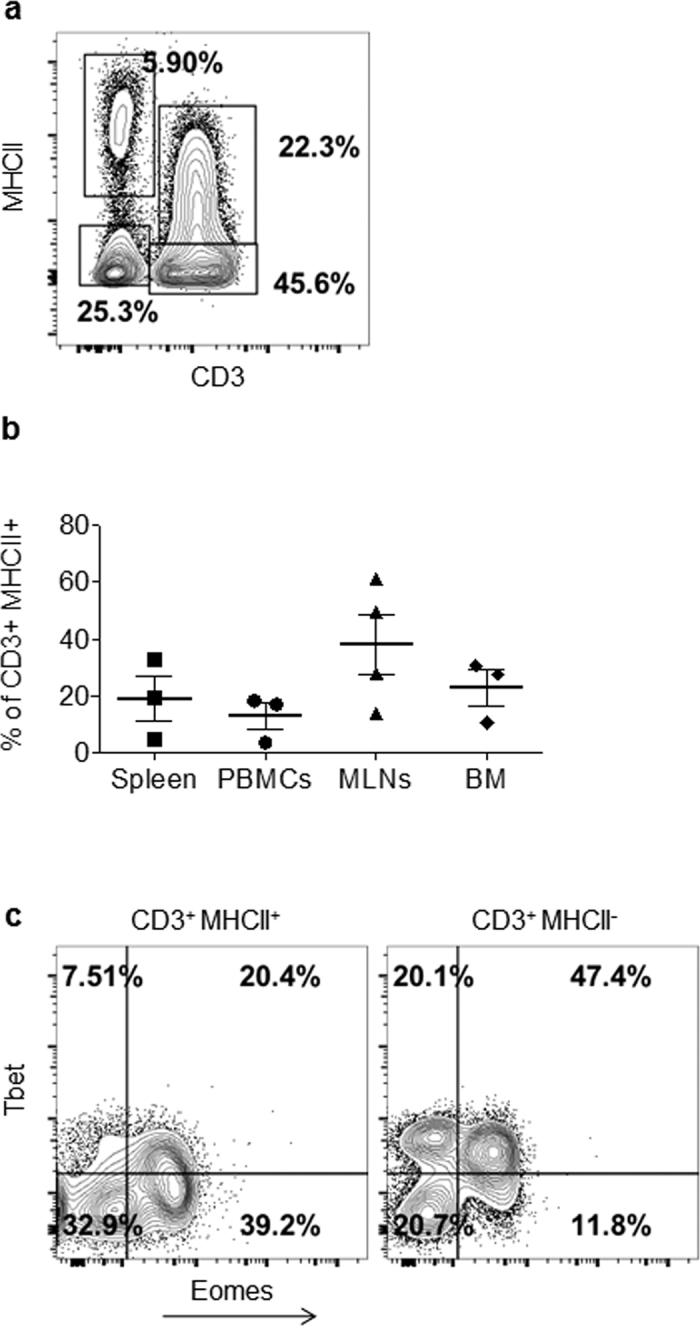
Expression of MHCII molecules by CD3^+^ T cells. (**a**) FACS analysis of splenocytes from one bat based on detection of MHCII and CD3 molecules. (**b**) Individual percentages of MHCII^+^ T cells in spleen, MLN, PBMCs and BM from 3–4 bats. The mean (horizontal bar) and SEM are shown. (**c**) Dot plot featuring Tbet and Eomes expression in MHCII^+^ and MHCII^−^ CD3^+^ splenocytes from one bat.

It has been well recognized that activated T cells from many species, including humans but not mice, synthesize and express MHCII molecules on their surface^36^. Consistently, a significant proportion of CD3^+^ T cells (22.3%) in *P. alecto* spleen was found to express MHCII (Fig. 4a). When looking at the MHCII^+^ T cells in various organs, higher percentages were detected in the MLN compared to bone marrow, spleen and PBMCs (Fig. 4b). Interestingly, the percentage of MHCII^+^ T cells varied greatly among bats (5–60% in the MLN for example), which likely reflects a differential activation status from one bat to another, as expected for wild caught animals.

Among the MHCII^+^ T cells, majority were Eomes^+^ single positive whereas majority of MHCII^−^ T cells were Eomes^+^ Tbet^+^ (CD8+ T cells) (Fig. 4c). However, these percentages varied between bats and between organs (data not shown).

### Detection of cytokines and cytolytic molecules upon mitogenic stimulation

**Figure 5:**
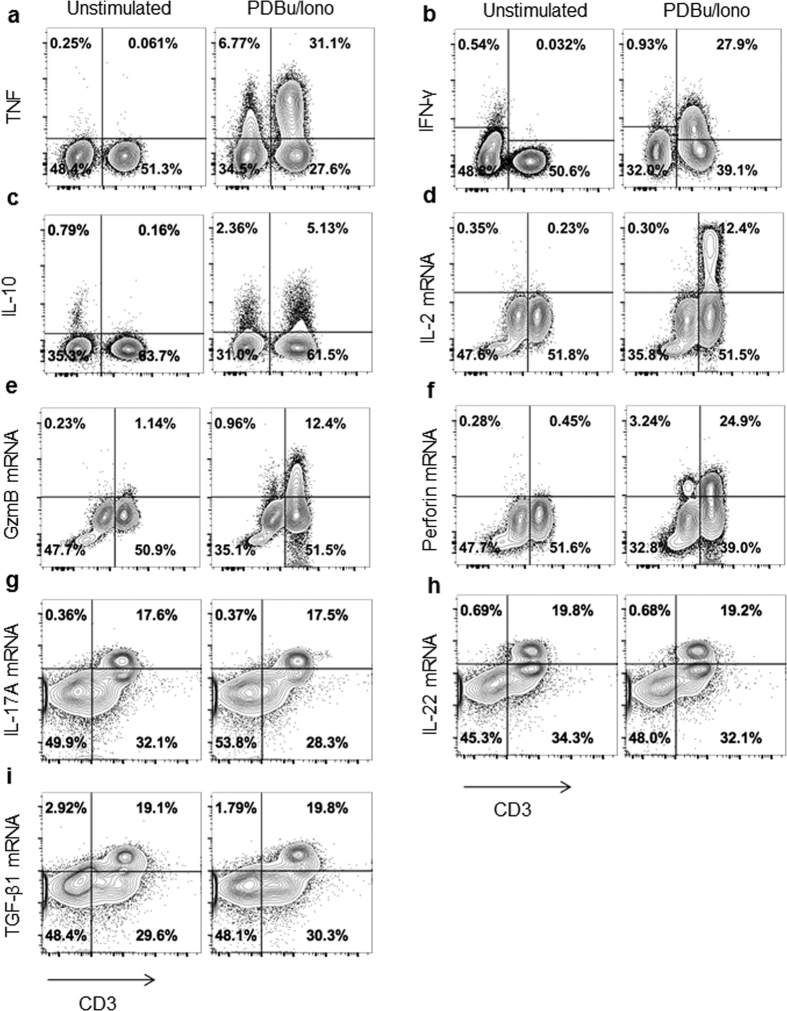
Bat T cell production of cytokines and cytolytic factors upon mitogenic stimulation. Bat splenocytes (**a–c,g–i**) or PBMCs (**d–f**) were stimulated for 4 h with PDBu/ionomycin or media in the presence of brefeldin A and monensin. Detection of intracellular TNF (**a**), IFN-γ (**b**), IL-10 (**c**) was performed at the protein level, and production of IL- 2 (**d**), granzyme B (**e**), perforin (**f**), IL-17a (**g**), IL-22 (**h**) and TGF-β1 (**i**) was detected at the mRNA level by Flow-FISH. Dot plots obtained with one bat are shown and are representative of the results obtained with 2–3 different bats.

To gain further insights into the functionality of *P. alecto* immune system, an *ex-vivo* mitogenic stimulation experiment was performed. Bat splenocytes or PBMCs were stimulated with PDBu and the ionophore ionomycin (activation of protein kinase C bypassing the T cell receptor), in the presence of brefeldin A and monensin that block the Golgi apparatus and allow intracellular accumulation of cytokines produced in response to the stimulation. Cross-reactive anti-human TNF and anti-human IL-10 antibodies, as well as anti-bat IFN-γ antibody were used to detect by flow cytometry the production of these cytokines. In addition, Flow-FISH was used to detect the production at the mRNA level of IL-2 (T cell growth factor), granzyme B and perforin (cytolytic molecules mainly produced by CD8^+^ T cells and NK cells^10^,^37^,^38^), IL-17A and IL-22 (inflammatory cytokines produced by Th17 cells) and transforming growth factor beta (TGF-β) (an immunosuppressive cytokine associated with Treg as well as Th17 differentiation^39^,^40^). Results showed that upon stimulation with PDBu/Iono a great proportion of the CD3^+^ cells present in the spleen produced TNF and IFN-γ (Fig. 5a,b), whereas a smaller percentage produced IL-10 (Fig. 5c). Expectedly, a greater percentage of CD3^+^ MHCII^+^ cells produced TNF compared to the MHCII^−^ T cell population (Fig. S2). Furthermore, TNF production in CD3^+^ T cell subsets ranged from 23.6% in Tbet^−^ Eomes^−^ Gata3^−^ (representing CD4^+^ T cells) to 73.5% in Tbet^−^ Eomes^+^ T cells (possibly representing CD8^+^ memory T cells) (Fig. S3a). However, those percentages varied greatly from one bat to another, again likely reflecting the differential activation status of immune cells in these wild-caught animals (Fig. S3b).

CD3^+^ cells producing IL-2, granzyme B and perforin mRNA were also detected in PBMCs stimulated with PDBu/Iono (Fig. 5d–f). Interestingly and unexpectedly, PdBu/Iono stimulation of splenocytes did not result in increased levels of IL-17A, IL-22 and TGF-β mRNA and instead constitutive expression of these mRNA was detected in ~40% of total CD3^+^ T cells (Fig. 5g–i). This observation may indicate a strong bias of CD4^+^ T cell towards the Th17 and Treg subsets in bat spleen in comparison to mouse and human spleen where the proportion of these subsets is considerably lower. Such hypothesis needs to be confirmed by the detection of IL-17, IL-22, and TGF-β at the protein level when specific antibodies are available.

**Figure 6:**
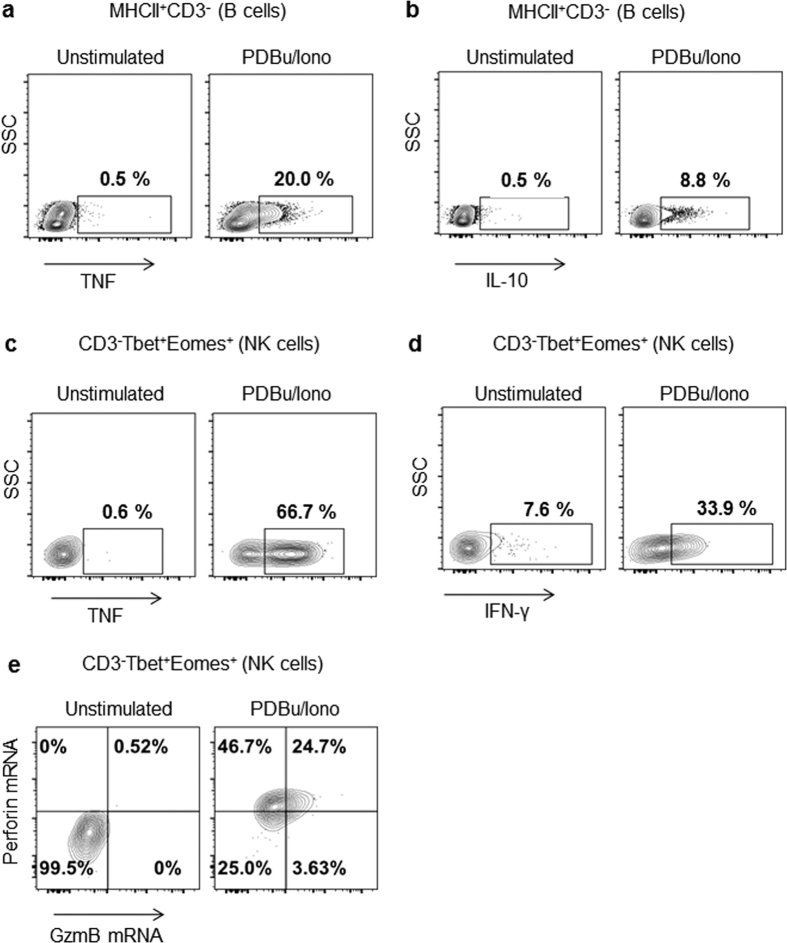
Production of cytokines by B and NK cells upon mitogenic stimulation. Bat splenocytes or PBMCs were stimulated for 4 h with PDBu/ionomycin or media in the presence of brefeldin A and monensin. Cells were gated on CD3^−^ MHCII^+^ (B cells) (**a,b**) or CD3^−^ Tbet^+^ Eomes^+^ (NK cells). Production of intracellular TNF (**a,c**), IL-10 (**b**), IFN-γ (**d**) was done at the protein level, and production of granzyme B and perforin (**e**) was performed at the mRNA level by Flow-FISH. Dot plots from one bat are shown and are representative of the results obtained with 2–3 different bats.

Both B cells and NK cells responded to PDBu/Iono stimulation (Fig. 6). Of the B cells, 20% and 8.8% produced TNF and IL-10, respectively (Fig. 6a,b). Of the NK cells, 66.7% and 33.9% produced TNF and IFN-γ, respectively (Fig. 6c,d). Furthermore, 46.5% of NK cells were also found to produce perforin and 24.7% produced both perforin and granzyme B (Fig. 6e).

Together, these results demonstrate for the first time that the main lymphocyte populations in bats are able to produce effector molecules such as cytokines and cytolytic factors in response to mitogenic stimulation.

### Proliferative capacity of bat T cells

**Figure 7:**
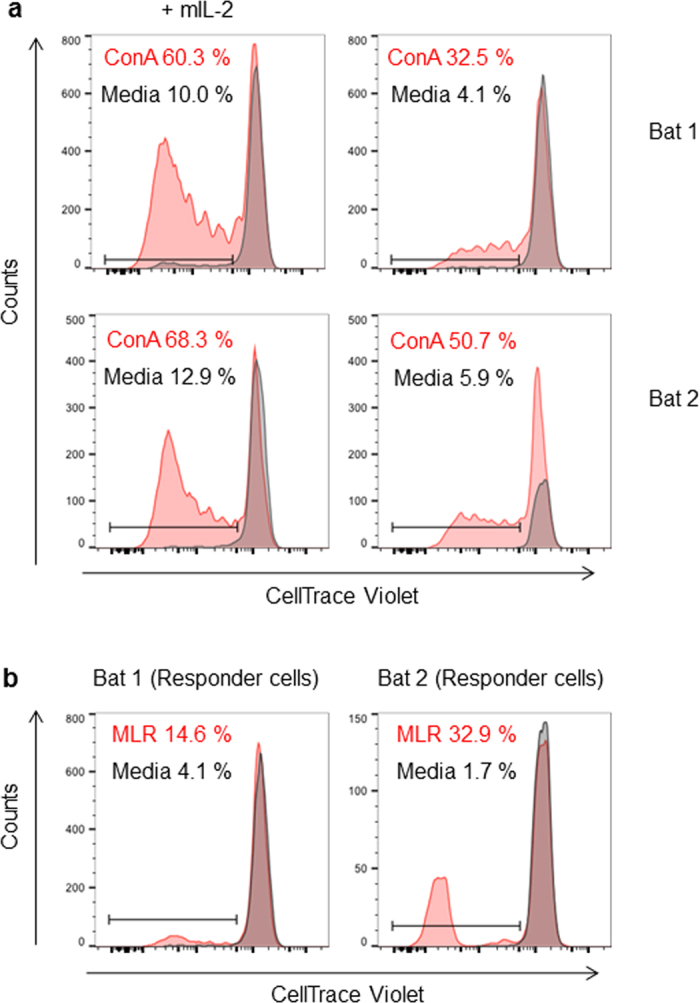
Proliferation of *P. alecto* T cells upon Concanavalin A stimulation. (**a**) Splenocytes from two different bats were stimulated with ConA (red) or media (grey) for 5 days in the presence or absence of mouse recombinant IL-2 (mIL-2). Cells were gated on CD3^+^. (**b**) Allogeneic Mixed Lymphocyte Reaction (MLR), “stimulator cells” (prepared form one bat spleen) were treated with Mitomycin C and were then co-cultured for 5 days with Cell Trace Violet-labeled “responder cells” (prepared from another bat spleen) (red) or were cultured alone in media (grey). Graphs shown represent cells gated on CD3^+^ cells. This experiment was repeated twice independently.

The proliferative capacity of bat immune cells was also evaluated. Bats splenocytes were labeled with Cell Trace Violet and stimulated for 5 days with Concanavalin A, a lectin with mitogenic activity, in the presence or absence of mouse recombinant IL-2 (mIL-2). In human and mouse, IL-2 has indeed been known to stimulate proliferation of responsive T cells^41^. Figure 7a shows representative histograms gated on CD3^+^ cells from two different bats. ConA-stimulated cells divided in the absence of mIL-2 as compared to the unstimulated cells (Fig. 7a). Interestingly, increased percentages of dividing cells were observed in the presence of mIL-2, suggesting that bat T cells are able to recognize and respond to murine IL-2.

An allogeneic mixed lymphocyte reaction (MLR) was also carried out where “splenocyte responder cells” from bat 1 (or bat 2) labeled with Cell Tracer Violet were incubated with “splenocyte simulator cells” from bat 2 (or bat 1) which were pre-treated with mitomycin, to inhibit proliferation. Proliferation of the “responder cells” after 5 days of co-culture was then measured by flow cytometry, gating on CD3^+^ cells. Significant proportions of the responder T cells proliferated compared to the media control, indicating the ability of cells from bat 1 to respond to the presence of cells from bat 2, and vice versa.

Together, these experiments demonstrate that bat T cells are able to proliferate in response to either mitogenic stimulation or to the presence of non-self antigens.

## Discussion

A handful of studies conducted in the 80 s and early 90 s described lymphocyte-like cells in *Pteropus* bats based on morphological evidence and also reported their delayed responses to mitogens as compared to mice^42^,^43^,^44^,^45^. More recently, with the genome sequences of a few bat species (including *P. alecto*) and some of their transcriptomes available^20^,^21^,^22^,^46^, *in silico* analyses have provided further insights into the genetic makeup of the bat’s immune system. In particular, the identification of immune-related transcripts linked to cytokine production, lymphocyte activation, regulation of apoptosis and regulation of lymphocyte activation were among the most represented transcripts found^20^,^22^. Transcripts associated with T and B cell identification, activation, inhibition and differentiation have been reported to be similar to other mammals. Interestingly, the absence of NK cell-related genes such as KLRs and CD16 in the transcriptome of various bat species suggests that the NK cell receptor repertoire in bats might be significantly different from that found in other mammals^20^,^22^,^46^.

Here, we provided the first phenotypic and functional characterization of major adaptive immune cell populations in the fruit-eating bat *P. alecto.* While awaiting the development of bat specific reagents, i.e. antibodies, we report the use of a combination of cross-reactive antibodies that allow the identification of various T cells subsets, B cells and NK cells. Furthermore, this work describes for the first time the detection of effector molecules not only at the mRNA but also at the protein level including cytokines and cytolytic factors upon mitogenic stimulation of bat lymphocytes and opens up possibilities for measuring functional aspects of bat immune cells.

A number of interesting observations were made and pave the way towards a better understanding of the possible immune mechanisms and strategies evolved by bats to control and tolerate viruses that in humans cause high mortality rate.

Firstly, our data revealed a different proportion of CD4^+^ and CD8^+^ T cells depending on the organ/tissue examined. In spleen, CD8^+^ T cells were predominant over the CD4^+^ T cell subsets. In the lymph nodes, a similar proportion of Eomes^−^ Tbet^−^ Gata3^−^ (CD4^+^ T cells) and T cells expressing Eomes only (which represents a CD8 memory phenotype in humans and mice) was observed. Instead, Gata3-expressing T cells were predominant in PBMCs suggesting that CD4^+^ T cells outnumber CD8^+^ T cells in the blood. Similarly, the bone marrow contained Tbet^+^ single positive cells (CD4^+^ Th1 cells) and Tbet^+^ Eomes^+^ double positive cells (CD8^+^ T cells) in a 2 to 1 ratio. Surprisingly, over 30% of leukocytes present in the bats bone marrow are T lymphocytes, a significantly greater percentage than that described in human and mouse bone marrow which ranges between 4% and 8%. Furthermore, the CD4^+^ to CD8^+^ T cell ratio in human and mouse bone marrow is reported to be 1 to 2^35^, as opposed to the 2:1 ratio found in bats. This unique T cell profile may suggest that the bat bone marrow assumes the function of the thymus in providing support for T cell development. Alternatively, on-going infection, inflammation and/or autoimmunity could explain the high number of CD4^+^ T cells in the bone marrow of these wild caught bats^47^,^48^,^49^,^50^. Likewise, predominance of CD8^+^ T cells in the spleen from these animals may indicate the presence of viruses in this organ or may represent the immune steady state of the bat spleen, specifically geared towards fighting viral infections. Interestingly, when total splenocytes from these bats were incubated with whole dengue virus (DENV) or peptides derived from various viruses including DENV, Nipah and rabies virus, no significant cytokine production and/or cell proliferation could be measured (data not shown). This result indicates that these bats have not been exposed to these viruses before, and may favor the hypothesis that at steady state, bats secondary lymhoid organs such as the spleen have a higher proportion of CD8^+^ T cells than other mammals. Further investigation in bats bred under specific pathogen free conditions in which an immune steady state or baseline can be established would be required to address these hypotheses.

Secondly, in all the organs examined, most of the B cells (CD3^−^ CD11b^−^ MHCII^+^) stained positive for IgG. In mouse and human, MHCII^+^ IgG-expressing B cells are B cells that have previously encountered and interacted with their cognate specific antigen, and undergone antibody isotype switching as part of their activation and differentiation process. They can be either antibody secreting cells (plasmablasts) or long lived memory B cells^51^,^52^. The fact that most of the bat B cells examined are IgG^+^ thus suggests that these cells are antigen-primed B cells that have undergone somatic hypermutation of their B cell receptor. This finding is not very surprising and likely reflects that these wild-caught bats have been exposed to antigens from various pathogens and other sources. Further characterization of bat B cells includes assessing the ability of these cells to present antigens and to respond to specific stimulation such as TLR agonists or anti-Ig antibodies, and their ability to secrete antibodies.

Thirdly, co-incubation of bat splenocytes with murine IL-2 (mIL-2), a well-known and important growth factor for T cells, led to greater proliferation of T cells, suggesting cross-reactivity between mIL-2 and its cognate bat receptor. This finding may have important implications for the expansion and maintenance of bat T cells in culture. The amino acid sequence homology between mouse and bat IL-2 is 53%. Interestingly, the IL-2R-α chain, also known as CD25, was not found or was not annotated in *P. alecto* genome. Nonetheless, the amino acid sequence homology of the other two IL-2 receptor chains IL-2R-β and IL-2R-γ, between mouse and bat reached 58% and 64% respectively.

Lastly, a significant proportion of CD3^+^ T cells in bat spleen was found to constitutively express IL-17A, IL-22 and TGF-β at the mRNA level. The co-expression of these factors is consistent with the fact that the development of Th17 cells and Tregs is closely related, with both cell types depending on TGF-β signaling^39^. It is worth noting that Th17 cells have been reported to play an active role in tumor regression which is tempting to link to the low level of tumor-related mortality reported in bats^53^. As for Tregs, their role is to suppress or negatively regulate virus-induced CD8^+^ T cell-mediated inflammation, in order to prevent extensive tissue damage. This is consistent with the predominance of CD8+ T cells found in bat spleen.

In conclusion, this study represents a landmark in the field of bat immunology being the first to describe a strategy for phenotypic and functional characterization of the main lymphocyte populations in bats.

## Materials and Methods

All the *ex vivo* biological experiments described were conducted in a Biosafety Level 2 containment facility and were approved by the institutional biosafety committee of National University of Singapore.

### Animals

*P. alecto* bats used in this study were caught in Queensland (Australia) and transported to the Australian Animal Health Laboratory (AAHL), CSIRO (Victoria, Australia). Only bats testing negative by qPCR for Hendra virus (HeV) and Lyssavirus were included in the study. The procedures performed were in accordance with AAHL Animal Ethics guidelines and approved by the AAHL Animal Ethics Committee (Protocol No. 1557). Three to five bat specimens were used in this study.

### *In silico* analysis

Sequences from *P. alecto* were obtained from the current genome assembly in the Ensembl database and sequences from *Homo sapiens* (human) and *Mus musculus* (mouse) were obtained from the NCBI database. *P. alecto* nucleotide sequences were translated into protein sequences using the Expasy Translate Tool program (http://web.expasy.org/translate/) and protein sequence alignment was performed using ClustalX program (http://www.ebi.ac.uk/Tools/msa/clustalo/).

### Sample processing for FACS analysis

Single cell suspension from spleen, mediastinal lymph nodes, bone marrow or blood (peripheral blood mononuclear cells, PBMCs) were stained with Fixable Viability Dye e780 (eBioscience) for 20 min at 4 °C, then 10% FBS was added and cells were incubated for another 10 min. For staining of surface markers, the cells were incubated first with primary antibodies (Table S2) including anti-mouse I-A/I-E MHC class II (clone 2G9, BD), polyclonal anti-bat IgG (Novus Biologicals, NB7237), anti-mouse CD11b (clone M1/70, eBioscience) and anti-mouse CD44 (clone IM7, BD) for 30 min at 4 °C. This was followed by incubation with fluorescently labeled anti-goat IgG secondary antibody (ThermoFisher) for 30 min at 4 °C. For staining of intracellular proteins (including transcription factors and cytokines), the cells were first fixed and permeabilized using the Fix/Perm Foxp3-staining kit (eBioscience) for 30 min at 4 °C. Staining was performed at room temperature in the dark with the following antibodies anti-CD3 (clone CD3–12, Abcam), anti-mouse Eomes (clone Dan11mag), anti-human/mouse Tbet (clone 4B10), and anti-human/mouse Gata3 (clone TWAJ) antibodies (Table S2). All antibodies were purchased from eBioscience unless otherwise stated. Samples were analyzed using LSR Fortessa X-20 (BD) and Flowjo software.

### Mitogenic cell stimulation

Single cell suspensions prepared in complete medium (RPMI + 10% FBS + 1 mM Sodium pyruvate) were incubated with 50 nM phorbol 12, 13-dibutyrate (PDBu) and 0.5 μg/ml ionomycin (Sigma) for 4 h before further processing including measurement of cytokine production by flow cytometry or Flow-FISH.

For intracellular cytokine detection monensin and brefeldin A (eBioscience) were added during the last 2 h of the stimulation. Staining was done as described in the previous section and the following antibodies were used: anti-human TNF (clone Mab11, BD), anti-human IL-10 (clone JES3–19F1, Biolegend) and anti-bat IFN-γ (clone 2G6^45^, a kind gift from CSIRO) + F(ab’)2 anti-mouse IgG PE-conjugated (eBioscience) (Table S2).

### PrimeFlow-fluorescence *in-situ* hybridization (Flow-FISH)

The FlowRNA I Assay kit (Affymetrix eBioscience) was used according to the manufacturer’s instructions^33^. Briefly, one million cells per sample were first stained with a live fixable viability dye (eBioscience), then fixed and permeabilized according to the manufacturer’s instructions. Cells were then incubated with anti- CD3, Tbet, Eomes and Gata3 antibodies for 30 min at RT, followed by *in-situ* hybridization with the Alexa Fluor 647-labelled CD4 and CD8 mRNA oligo probes (1 μM final concentration for each oligo, Table S3). Analysis was done using LSRFortessa X-20 (BD) and Flowjo software. This technology was also performed after 4 h re-stimulation with PDBu/iono to stain for IL-2, Granzyme B, Perforin, IL-17A, IL-22 and TGF-β1 mRNA using Alexa Fluor 647, Alexa Fluor 488 or Alexa Fluor 750-labelled oligo probes (Table S3).

### Proliferation and Mixed Lymphocyte Reaction

Splenocytes (1 × 10^7^/ml in PBS) were incubated with Cell Trace Violet (Invitrogen) (0.5 μM final concentration) for 20 min at 37 °C and 5% CO_2_, then 10% FBS was added to the cells and incubated for 5 min on ice. After two washes with PBS supplemented with 2% FBS the cells were resuspended in culture media, seeded at 5 × 10^5^ cells per well in a 96-U bottom plate and cultured for 5 days at 37 °C and 5% CO_2_ with either 2 μg/ml concanavalin A (ConA) (Sigma) or media alone, with or without 20 U/ml recombinant mouse IL-2 (eBioscience).

For MLR, splenocyte suspensions were prepared from 2 different bats. A fraction from each preparation was treated with 50 μg/ml Mitomycin C for 1 h (stimulator cells), then washed and co-cultured at a 1:1 ratio for 5 days at 37 °C and 5% CO_2_ with 5 × 10^5^ cells previously labeled with Cell Trace Violet from the other bat preparation (responder cells). After 5 days incubation, cells were stained with specific antibodies and analyzed by flow cytometry (as described above).

## Additional Information

**How to cite this article**: Martínez Gómez, J. M. *et al*. Phenotypic and functional characterization of the major lymphocyte populations in the fruit-eating bat *Pteropus alecto. Sci. Rep.* **6**, 37796; doi: 10.1038/srep37796 (2016).

**Publisher's note:** Springer Nature remains neutral with regard to jurisdictional claims in published maps and institutional affiliations.

## Supplementary Material

Supplemental Dataset

## Figures and Tables

**Table 1:** Percentage of amino acid identity between proteins from *P. alecto* and human or mouse orthologs.

	Marker	Human	Mouse
Surface molecules	CD3ε	73	71
	CD4	59	50
	CD8α	61	44
	CD19	68	63
	CD11b	78	75
Transcription factors	Tbet	94	88
	Eomes	94	90
	Gata3	95	95
	Foxp3	89	86
	RORγt	87	86
Cytokines	IL-2	66	53
	IL-10	83	75
	TNF	88	80
Cytolytic factors	Perforin	77	70
	Granzyme B	52	53

ClustalX program was used for protein alignment between *P. alecto* sequences (genome data obtained from the Ensembl database) and sequences from *Homo sapiens* (human) and *Mus musculus* (mouse).
