# Supplementary material for: Phenotypic and functional characterization of the major lymphocyte populations in the fruit-eating bat Pteropus alecto
Source: Sci Rep. 2016 Nov 24;6:37796. doi: 10.1038/srep37796 (PMC5121612; doi:10.1038/srep37796)
Supplement: Supplemental Dataset [file srep37796-s1.doc]

**Supplemental Information**

**Full title: Phenotypic and functional characterization of the major lymphocyte populations in the fruit-eating bat *Pteropus alecto***

**Authors:** Julia María Martínez Gómez^1,2^, Pravin Periasamy^1,2^, Charles-Antoine Dutertre^3,4^, Aaron Trent Irving^3^, Justin Han Jia Ng^3^, Gary Crameri^5^, Michelle L. Baker^5^, Florent Ginhoux^4^, Lin-Fa Wang^3^ and Sylvie Alonso^1,2*^

**Affiliations:** ^1^Department of Microbiology and Immunology, Yong Loo Lin School of Medicine, National University of Singapore, Singapore. ^2^Immunology programme, Life Sciences Institute, Yong Loo Lin School of Medicine, National University of Singapore, Singapore. ^3^Programme in Emerging Infectious Disease, Duke-NUS Medical School, Singapore. ^4^Singapore Immunology Network (SIgN), Agency for Science, Technology and Research (ASTAR), Singapore. ^5^CSIRO, Health and Biosecurity Business Unit, Australian Animal Health Laboratory, Geelong, Australia.

*Corresponding author: Immunology programme, CeLS building, 28 Medical Drive, NUS, Singapore 117456. Tel: +65 65163541; Email: [micas@nus.edu.sg](mailto:micas@nus.edu.sg); Fax: +65 67782684.

**Supplemental Figure 1**

**Figure S1. Detection of CD11b, MHCII and IgG in bat T cells.** Bat PBMCs were stained with cross-reactive antibodies and analyzed by FACS. Dot plots shown here are gated on live cells, singlets and within the lymphocyte region. One set of data obtained from one bat is shown, and is representative of 3 different bats analyzed.

**Supplemental Figure 2**

**Figure S2. TNF production by MHCII^+^ and MHCII^-^ CD3^+^ T cells.** Splenocytes were stimulated with PDBu/Ionomycin (bottom) or media (top), and detection of intracellular TNF in MHCII^+^ (left dot plot) or MHCII^-^ (right dot plot) CD3^+^ T cells is shown. Dot plots shown are from one representative bat out of 3 different bats analyzed.

**Supplemental Figure 3**

**Figure S3. TNF production by T cell subsets upon mitogenic stimulation.** Splenocytes were stimulated with PDBu/Ionomycin or media, and detection of intracellular production of TNF in various CD3^+^ T cells subsets based on the differential expression of transcription factors was carried out. Dot plots from one representative bat (a) are shown. (b) Results obtained in 3 bats. Each bat is represented by a different symbol.

**Table S1.** Human and mouse-specific antibodies assayed for detection of surface markers and intracellular molecules in *P. alecto* splenocytes*.* Successful cross-reactive antibodies are indicated in bold.

| **Target** | **Clone** | **Species**  **Reactivity** |
| --- | --- | --- |
| CD3 | 145-2C11 | mouse |
| CD3 | UCHT1 | human |
| CD3 | SK7 | human |
| CD3 | OKT3 | human |
| CD3 | 17A2 | mouse |
| CD3 | KT3 | mouse |
| CD3 | 5B2 | m/r/h |
| CD3 | RM0027-3B19 | mouse |
| CD3 | 21-L5 | human |
| **CD3** | **CD3-12** | **most mammals** |
| CD4 | RM4-5 | mouse |
| CD4 | OKT4 | human |
| CD4 | H129.19 | mouse |
| CD4 | GK1.5 | mouse |
| CD4 | RIV6 | m/r/h |
| CD4 | MEM-115 | human |
| CD4 | 5B4 | human |
| CD4 | Polyclonal | many species |
| CD8 | RPA-T8 | human |
| CD8 | UCHT4 | human |
| CD8 | 32-M4 | human |
| CD8 | HIT8a | human |
| **CD11b** | **M1/70** | **m/h/primates/rabbit** |
| CD19 | LT19 | human |
| CD19 | ID3 | mouse |
| CD19 | SJ25C1 | human |
| CD19 | HD237 | human |
| CD19 | 6D5 | mouse |
| CD19 | MB19-1 | mouse |
| **CD44** | **IM7** | **many species** |
| NK1.1 | PK136 | human |
| NK | DX5 | mouse |
| CD56 | Polyclonal | many species |
| CD56 | MEM-188 | human |
| NKp46 | 29A1.4 | mouse |
| NKp44 | P44-8 | human |
| MHCII | M5/114.15.2 | mouse |
| **MHCII** | **2G9** | **mouse** |
| Foxp3 | 150D/E4 | m/r/h |
| Foxp3 | FJK-16S | m/r |
| RORγt | AFKJS-9 | h/m |
| Bcl6 | BCL-DWN | h/m |
| **Tbet** | **4B10** | **h/m** |
| **Gata3** | **TWAJ** | **h/m** |
| **Eomes** | **Dan11mag** | **mouse** |
| **TNF** | **Mab11** | **human** |
| **IL-10** | **JES3-19F1** | **human** |

**Table S2.** Antibodies used for surface and intracellular staining.

| **Marker** | **Fluorophore** | **Clone** | **Concentration** | **Company** |
| --- | --- | --- | --- | --- |
| Anti-mouse I-A/I-E (MHC-II) | PE | 2G9 | 1 µg/ml | BD Bioscience |
| Anti-mouse I-A/I-E (MHC-II) | FITC | 2G9 | 2.5 µg/ml | BD Bioscience |
| Anti-mouse CD11b | e450 | M1/70 | 1 µg/ml | eBioscience |
| Anti-mouse CD11b | APC e780 | M1/70 | 1 µg/ml | eBioscience |
| Anti-mouse CD44 | V500 | IM7 | 1 µg/ml | BD Bioscience |
| Anti-bat IgG | unlabelled | polyclonal | 1 µg/ml | Novus Biologicals |
| Anti-goat IgG | A647 | polyclonal | 7 µg/ml | ThermoFisher |
| Anti-human CD3 | FITC | CD3-12 | 0.5 µg/ml | AbD Serotec |
| Anti-human CD3 | PB | CD3-12 | 0.25 µg/ml | AbD Serotec |
| Anti-human/mouse Tbet | PE-Cy7 | 4B10 | 0.7 µg/ml | eBioscience |
| Anti-mouse Eomes | PE-e610 | Dan11mag | 0.7 µg/ml | eBioscience |
| Anti-human/mouse Gata3 | PE | TWAJ | 2 µl per test | eBioscience |
| Anti-human/mouse Gata3 | APC | TWAJ | 2 µl per test | eBioscience |
| Anti-bat IFNγ | unlabelled | 2G6 | 1 in 10 dilution from stock | Kind gift from CSIRO |
| Anti-mouse IgG | PE | polyclonal | 2.5 µg/ml | eBioscience |
| Anti-human TNF | BUV395 | Mab11 | 5 µl per test | BD Bioscience |
| Anti-human IL10 | APC | JES3-19F1 | 2 µl per test | Biolegend |

**Table S3:** Probes sequences designed for Flow-FISH.

| **TARGET GENE (ACCESSION NUMBER)** | **PROBE BINDING REGION** | **SEQUENCE** |
| --- | --- | --- |
| *P. alecto* CD4 | 199-217bp | agcagcaggagcaagtgcc |
|  | 218-235bp | gggagaagcgccagttgc |
|  | 236-258bp | cttcttttccttgagtgatggct |
|  | 259-277bp | cctgccttacccagcacca |
|  | 278-298bp | cagggcagctctgctttgtct |
|  | 299-318bp | tcttcttctgggaagcctgg |
|  | 319-339bp | atttccagctgaagctcatgc |
|  | 340-362bp | tctcagaaccatgatcccagaat |
|  | 363-382bp | gccggaaaggagttcgaaga |
|  | 383-407bp | ggaacctatcatcaagaatgttgat |
|  | 408-428bp | aacacgtgttttcagccagga |
|  | 429-451bp | tcccatagggttttctttgattc |
|  | 452-472bp | accagaggaaaggatccttgg |
|  | 473-496bp | tctttcatgtcaagatccttgatg |
|  | 497-521bp | cacttcacagatgtaaatccctgag |
|  | 522-544bp | tccacctcttttgtcttgtcctc |
|  | 545-567bp | cattcaatctgaacaccagcaat |
|  | 568-586bp | ccgcggatgtccaaatcag |
|  | 587-605bp | catcaggtggatgctgcca |
|  | 606-625bp | agggtcagtctctccccagg |
|  | 626-643bp | ggcgggctctccaaggtc |
|  | 644-667bp | catactattgaagggttgctacca |
|  | 668-691bp | tactttttactccctggacctttc |
|  | 692-713bp | tgagaggctcttgtccccatta |
|  | 714-732bp | gccaccctagctgggacag |
|  | 733-754bp | cattcccaggtaccactctcct |
|  | 755-777bp | tcttcttgctgtaggagacgatg |
|  | 778-800bp | gatgtttatgctgagcaccagtg |
|  | 801-818bp | ccggatagccagcaccaa |
|  | 819-842bp | tgcatagactgtgttggagacctt |
|  | 843-862bp | tccaccttctccccctcttt |
|  | 863-884bp | aaaggtaagtgggaaggagagc |
|  | 885-907bp | tcaccttccaggttttcatcttc |
|  | 1184-1203bp | gcttgggggatctgagtccc |
|  | 1204-1225bp | tccagcttcaagctcagtgtca |
|  | 1226-1247bp | tgagaccttcgcagtctggttc |
|  | 1248-1269bp | ccactagctgcttctgctgttc |
|  | 1270-1287bp | cctcagggtccggcgtct |
|  | 1288-1309bp | aatagacactgccatgtccctg |
|  | 1310-1332bp | gcaggactttgtcctcgtcactc |
|  | 1333-1356bp | acagaacttcagtcttggattcca |
|  | 1357-1376bp | ggcctgagtgaaacctgcag |
|  | 1377-1395bp | tgaccaggagctttggcca |
|  | 1396-1413bp | ggatcccccccagcacaa |
|  | 1414-1434bp | cggtgaaaatcagaaagccca |
|  | 1435-1456bp | acacagcagaagatgcagatcc |
|  | 1457-1472bp | gcggtgccggcacttg |
|  | 1473-1489bp | cgctctgcctggcgctt |
|  | 1490-1515bp | tgaggagtcttttgatctgagacatc |
|  | 1516-1537bp | cactggcaggtcttcttctcac |
| *P. alecto* CD8 | 1417-1437bp | gcaggagacaacgaaatacgg |
|  | 1438-1458bp | gctcagacgcgagtagaacgc |
|  | 1459-1475bp | gcgcggctcagctttca |
|  | 1476-1490bp | tggcgcacccctgcg |
|  | 1491-1507bp | ggtcaccggcaaggcca |
|  | 1508-1523bp | ggggcaggagcagcca |
|  | 1524-1540bp | agcttgcagcagggcca |
|  | 1541-1557bp | tgcccgaaggtcgtggc |
|  | 1558-1576bp | cgacatccggaacgacagc |
|  | 1577-1597bp | caggtgactctgccctttctg |
|  | 1598-1615bp | cagctccacctgcttgcc |
|  | 1616-1633bp | cagcagcacttcgcagcg |
|  | 1634-1649bp | ccgacgccaggctgga |
|  | 1650-1669bp | ctgaaagagccacgagcagc |
|  | 1670-1685bp | tggcggctccaggagg |
|  | 1686-1707bp | tacattaggaagacagggctgg |
|  | 1708-1728bp | ttgatccggattttggagatg |
|  | 1729-1744bp | cagcccctcggccgtc |
|  | 1745-1765bp | acccgacatctgtttgctgtt |
|  | 1766-1786bp | gacggtgtcctggatcctctg |
|  | 1787-1805bp | tgtgcagggtgaggctgaa |
|  | 1806-1824bp | tgctcctcttcgcggaagt |
|  | 1825-1847bp | caaccgagcagaaatagtagcct |
|  | 1848-1873bp | gctgaagtacagtatcgagttgccta |
|  | 1874-1891bp | gaagacgggcacgaaggg |
|  | 1892-1909bp | ggtgggtgtcgctggcag |
|  | 1910-1923bp | ggcgcgggcgtcgt |
|  | 1924-1942bp | gactcgcgtgagtggtcgc |
|  | 1943-1960bp | ctgcgatgctttggtggg |
|  | 1961-1978bp | cgggctcagggacactgg |
|  | 1979-1994bp | ccggccggcacacctc |
|  | 1995-2009bp | ctgcgctgcccgctg |
|  | 2010-2027bp | gcagctccctcgtgtccg |
|  | 2028-2051bp | agatgtagatcacacaggcgaagt |
|  | 2052-2067bp | ccagccagaggcgccc |
|  | 2068-2088bp | aggagaaggaccacgcagatc |
|  | 2089-2112bp | cagatgatcgtgatgaccagagat |
|  | 2113-2133bp | cgtcttcggttcctgtgattg |
|  | 2134-2153bp | gcctgggacatttgcaaaca |
|  | 2154-2171bp | ctcctggtcggacgacgg |
|  | 2172-2188bp | tgaagggctcggcttgc |
| *P. alecto* IL-2 | 2-28bp | tgaacaagaagagcaatttatactgtt |
|  | 29-49bp | gagaggctgatgggaagctct |
|  | 50-77bp | gtcttgaggttactgtgagtagtgatta |
|  | 78-97bp | catcttgtgcatcgtggcag |
|  | 98-122bp | ttagtgcaatgcaagacaagaagta |
|  | 123-142bp | gtctgccaccagtgcaagag |
|  | 143-166bp | cctagagcttgaagtaggtgcacc |
|  | 167-188bp | ccagctgttgctgtgtttcctt |
|  | 189-212bp | gctgtaagtccttcagcaaatgct |
|  | 213-238bp | gtaattattaactgtctccaaaagcc |
|  | 239-260bp | tgctggagagttcgtgattctt |
|  | 261-284bp | gcatgtaaaatttaaaggtgagca |
|  | 285-306bp | tgattcagttctgtggcattgg |
|  | 307-325bp | gtccacgaggcactgcaga |
|  | 326-348bp | acttcctccaaaggtttgagttc |
|  | 349-374bp | agtttttgctttgagctatagttagc |
|  | 375-399bp | actgactccttgatgtctgagtgag |
|  | 400-423bp | tgagctgttacgttgatattgctc |
|  | 424-447bp | tttgtttcaggtccctttagtttc |
|  | 448-475bp | gctctcatcattatagacacatgtgtat |
|  | 476-498bp | ttcagaaattctttaacgttcgc |
|  | 499-522bp | ctttgacaaaaggtaatccatttg |
|  | 523-544bp | ttaagccagtgtcgagaggatg |
|  | 545-568bp | tgatacattttaaatgggagggac |
|  | 569-600bp | aatataatttttaaatatttaaataaatagcc |
|  | 601-627bp | agatagcaaaccatacatcaaaaaata |
|  | 628-655bp | tccgaagactaagaataatagttacaaa |
|  | 656-681bp | gaaccttaaaagatccatagtcatca |
| *P. alecto* Granzyme B | 2-26bp | gctgtccttctgtgatctcctttag |
|  | 27-46bp | ctgctgatgtcggagctgct |
|  | 47-66bp | gccacatcttcccaggaagg |
|  | 67-86bp | tgagagcaggagcaggaagg |
|  | 87-106bp | gagggcagtaaaaaggccag |
|  | 107-126bp | tctttcctgcctctgtccca |
|  | 127-144bp | cctcgtgtcccccgatga |
|  | 145-160bp | cgggagtggggcttgg |
|  | 161-182bp | ctgaatgtaggccatgtaaggg |
|  | 183-204bp | tcttatgcccattttgcagaaa |
|  | 205-222bp | ggacaccgccgcatctgt |
|  | 223-242bp | aacaaaattctcccgcacga |
|  | 243-264bp | cactgcagtgagcagctgtcag |
|  | 265-287bp | cagggtgacattgattgagcttc |
|  | 288-307bp | ttcttgatgttatgggcccc |
|  | 308-327bp | cttgctgggtcttctcctgc |
|  | 328-347bp | ggctttcaccacacggaaga |
|  | 348-369bp | gattatagtctgggtgggggat |
|  | 370-395bp | catgatgtcattggagaagttcttag |
|  | 396-420bp | tgatatttttctccagctgcagtaa |
|  | 421-441bp | gcttcacagctttcgtcagct |
|  | 442-457bp | ctgggcaggcggagga |
|  | 458-476bp | catcacccggtcctcttcc |
|  | 477-498bp | ccacactgcacttctgtcctgg |
|  | 499-512bp | ccgcccccaaccgg |
|  | 513-535bp | gggtatgtgtttatcagggcgat |
|  | 536-556bp | tccacctcctgcagtgtgtct |
|  | 557-576bp | aatccttttgcacggtcagc |
|  | 577-598bp | cgtaagtggcattcacactccc |
|  | 599-623bp | ttgaatggcactgttgtaataattt |
|  | 624-642bp | tggggttccccacacacag |
|  | 643-663bp | gaaaggaagccttgttctccg |
|  | 664-679bp | ccgccagagtcgccct |
|  | 680-706bp | tggatcacattcttacatactagaggg |
|  | 707-727bp | cgtccataggagacaatgccc |
|  | 728-746bp | cggagacgtcccgtttttt |
|  | 747-768bp | ttgagactttagtgtaggcccg |
|  | 769-792bp | ttttctttatccaggacaggaatt |
| *P. alecto* Perforin | 675-696bp | cttcttgaaattggggtggagt |
|  | 697-713bp | ggcagggccctgacagc |
|  | 714-732bp | ggaggtgttgaagtcgggg |
|  | 733-757bp | tgagcctgaagtagtcagactctgt |
|  | 758-781bp | tgaaatgggtaccataattggaga |
|  | 782-799bp | ccagttccatggagcgga |
|  | 800-815bp | gccgagatctggccgc |
|  | 816-834bp | ggtacgcagggcggtaagg |
|  | 835-851bp | tccagggccatctcgca |
|  | 852-871bp | cctcgtcagctgtgagccct |
|  | 872-888bp | ggccaggcagtctccca |
|  | 889-908bp | atgctgacctcagcctcgac |
|  | 909-926bp | gagctggcaaggccacct |
|  | 927-947bp | caggccttgattttcgatgag |
|  | 948-970bp | tgagctgcttcttcttctcctca |
|  | 971-993bp | ctggtggaaggagtctgtcatct |
|  | 994-1012bp | agtagcgttcccggtaggc |
|  | 1013-1033bp | ggtgaccgcctactacttcgg |
|  | 1034-1053bp | caggtcatgcacggaggtgt |
|  | 1054-1072bp | cagcgtggttcccgaacaa |
|  | 1073-1092bp | ggctgagaactgctcaggcc |
|  | 1093-1108bp | gcagcgaggccaccca |
|  | 1109-1128bp | cactaggctggggctgtcct |
|  | 1129-1153bp | catgcagaggctctagagtgtagtc |
|  | 1154-1173bp | gtcctggctctccaagagca |
|  | 1174-1188bp | tgcctcccgccgtgg |
|  | 1189-1207bp | tgctcacggcttgcctcag |
|  | 1208-1227bp | tgccctgcgcatcacatact |
|  | 1228-1245bp | gctgcagtccctccagcg |
|  | 1246-1260bp | tggagggcagggccg |
|  | 1261-1278bp | ggggctcttctgctgccc |
|  | 1279-1297bp | cacactggcaggggtcgtg |
|  | 1298-1315bp | cccctgagccatggcaca |
|  | 1316-1335bp | acagcagtccttgtcggtgg |
|  | 1336-1351bp | agcccctcttccgggg |
|  | 1352-1369bp | taacctccagctcggcca |
|  | 1370-1389bp | accactggcctggaagttca |
|  | 1390-1412bp | gcagtgaatatgtctccccacag |
|  | 1413-1434bp | gaccttcacataggcgtctgtg |
|  | 1435-1454bp | cgttcctggcctccaaagaa |
| *P. alecto* IL-17A | *43-64bp* | catgaatgaagttcccacagga |
|  | *65-85bp* | cagcagcagcagtgactggaa |
|  | *86-106bp* | ccctatggccaccagactcag |
|  | *107-127bp* | tgggattgctattcctgcctt |
|  | *128-150bp* | gtatttgagcatcctggattttg |
|  | *151-173bp* | tctgagggaagttcttgtcctca |
|  | *174-197bp* | ggatgtttaggttgaccttcacag |
|  | *198-220bp* | tctgagattcgtattccggttaa |
|  | *221-243bp* | cgattgtaataatccgaaggcct |
|  | *244-265bp* | gagattccaaggtgaggtggat |
|  | *266-284bp* | cagggtcctcattgcggtg |
|  | *285-308bp* | cccagatcacagagggatatctct |
|  | *309-324bp* | tggcggcacttcgcct |
|  | *325-347bp* | cttcaccattaacgcagaacaag |
|  | *348-369bp* | ttcaggtggtggtctaccttcc |
|  | *370-389bp* | cttgctgaatgggaacggag |
|  | *390-409bp* | tcttcgcaggaccaggatct |
|  | *410-426bp* | gggcagtgccgaggctg |
|  | *427-449bp* | tcttttccatctggaaggagttg |
|  | *450-469bp* | gcagccgacattcactagca |
|  | *470-490bp* | gacaatgggggtgacacaggt |
|  | *491-511bp* | aaattcttaagccacatggcg |
|  | *512-530bp* | gaatctggggtcaggctgg |
|  | *531-552bp* | cccagaaagcctactgatttgg |
|  | *553-578bp* | ggttagaaatgagactgggtctactc |
|  | *579-606bp* | gaacttgtttaaaaatcctagtgagttt |
|  | *607-634bp* | tgaattatctttgaaatttgaattaaat |
|  | *635-662bp* | aatctctgaatgaaatacctctgtatct |
|  | *663-687bp* | ggagaggaaaagaagattcagattc |
|  | *688-711bp* | ctcagtttaaaccttcttccttgg |
|  | *712-734bp* | agcaaacaagaagcaaattggta |
| *P. alecto* IL-22 | *2-23bp* | aacagttggtggctagggaaga |
|  | *24-47bp* | gccattgcagacaactctaactca |
|  | *48-64bp* | cagatttccgcggggtg |
|  | *65-87bp* | agtcctcataagggaaaagctca |
|  | *88-105bp* | aaggcagccagtggccag |
|  | *106-125bp* | accgacagggcaatgagaag |
|  | *126-145bp* | gcacagctgctccttcctgc |
|  | *146-166bp* | gtctgcagagagacccaatgg |
|  | *167-188bp* | tgctggaagttggacttgtcaa |
|  | *189-209bp* | gtgtggttggtgatgtagggc |
|  | *210-230bp* | gcctctttagccagcatgaaa |
|  | *231-253bp* | ctgtattgttgtctgccgaccta |
|  | *254-274bp* | tctccccaatgagacgaacat |
|  | *275-297bp* | catatggactccacggaacagtt |
|  | *298-319bp* | tcatcagatagcagcgctcact |
|  | *320-341bp* | agggtaaagttcagcacctgct |
|  | *342-361bp* | gggggagcaacacttcttca |
|  | *362-385bp* | tgtagggctggaatctatcagact |
|  | *386-406bp* | ggaaaggcactacttcctgca |
|  | *407-424bp* | tgttgctgagcctggcca |
|  | *425-450bp* | actcttaatatgacattggcttagct |
|  | *451-475bp* | cgtttctttggatatgctgatcatc |
|  | *476-497bp* | actgtgtccttcagcttttgca |
|  | *498-520bp* | ctccactctctccaagctttttc |
|  | *521-545bp* | tctagttctccaattgctttgattt |
|  | *546-568bp* | ttctcagggtcataaacagcaagttt |
|  | *569-591bp* | ttgctgtgatcaaatgcagacat |
|  | *592-617bp* | gaagtgttagtattcattttccagct |
|  | *618-645bp* | ggatcttattattatttctaacaggcaa |
|  | *646-669bp* | gggagaaaaaaaaaaaacctatgg |
|  | *670-689bp* | ggcttcccactttccttttg |
|  | *690-718bp* | tttagaatctacctatgattgtgaagttt |
|  | *719-741bp* | tttttaagctaagcaggggttta |
|  | *742-766bp* | atggacacaagtgacattgttctct |
|  | *767-791bp* | actaccaggactaccttctggtctt |
|  | *792-816bp* | aggaacaaatctcattatgaccaga |
| *P. Alecto* TGFβ1 | *862-874bp* | gggcagcgggccg |
|  | *875-892bp* | aagggccagcacggactc |
|  | *893-913bp* | ccggtcacgggtactgttgta |
|  | *914-929bp* | cgctctccccggccac |
|  | *930-945bp* | tcgggctccggctctg |
|  | *946-965bp* | cgtagtagtccgcctccggt |
|  | *966-983bp* | cgcgggtgacctccttgg |
|  | *984-1004bp* | gggtgttttccacggttagca |
|  | *1005-1029bp* | tgcttgtatgtctcgtagattttgt |
|  | *1030-1057bp* | gaagaacatgtatatgctgtgtgaatta |
|  | *1058-1075bp* | ccggagctcggacgtgtt |
|  | *1076-1093bp* | gggttccgacaccgcttc |
|  | *1094-1113bp* | tctgctcgggagagcaactc |
|  | *1114-1132bp* | gtgcctctgcatacgcagc |
|  | *1133-1154bp* | catgctgctccactttcaattt |
|  | *1155-1177bp* | gctgtatttctggtacagctcca |
|  | *1178-1196bp* | ggtagcgccaggaaccgtt |
|  | *1197-1215bp* | gccaggagctggttgctga |
|  | *1216-1231bp* | aggcgtgtcgctgggg |
|  | *1232-1253bp* | tgacctcaaaggacagccactc |
|  | *1254-1273bp* | ccactgccgcactactccag |
|  | *1274-1295bp* | caatttcccctttgtgactcag |
|  | *1296-1314bp* | gcactgaggcgaaagccct |
|  | *1315-1336bp* | tttgttgtcacaggagcagtgg |
|  | *1337-1362bp* | ttgatttccacatgaagtatgtcatc |
|  | *1363-1382bp* | ggcggtccgtactgattcca |
|  | *1383-1399bp* | ggtggccaggtctcccc |
|  | *1400-1419bp* | ggccggtttatgccatgaat |
|  | *1420-1438bp* | ggccatgaggagcaggaag |
|  | *1439-1457bp* | gggctctctccagaggggt |
|  | *1458-1476bp* | cgggaactgtgcaggtgct |
|  | *1477-1491bp* | agggctcggcggtgc |
|  | *1492-1513bp* | gctgaagcagtagttggtgtcc |
|  | *1514-1534bp* | acagcagttcttctccgtgga |
|  | *1535-1554bp* | tcaatgtaaagctgccgcac |
|  | *1555-1572bp* | cccaggtccttgcggaag |
|  | *1573-1592bp* | gctcgtggatccacttccag |
|  | *1593-1610bp* | tggcatggtagcccttgg |
|  | *1611-1627bp* | gggcccgaggcagaagt |
|  | *1628-1648bp* | caggctccaaatgtagggaca |
